# Baseline incidence of meningitis, malaria, mortality and other health outcomes in infants and young sub-Saharan African children prior to the introduction of the RTS,S/AS01_E_ malaria vaccine

**DOI:** 10.1186/s12936-021-03670-w

**Published:** 2021-04-26

**Authors:** The RTS,S Epidemiology EPI-MAL-002 Study Group, Prince Darko Agyapong, Elaine Jacqueline Akite, Nana Akosua Ansah, Patrick Odum Ansah, Kwaku Poku Asante, Denis Azabra Awuni, Daniel K. Azongo, Owusu Boahen, Marie-Cecile Bozonnat, Nathanial K. Copeland, Yolanda Guerra Mendoza, Valerie Haine, Samuel Bernard Ekow Harrison, Seyram Kaali, Michael Bandasua Kaburise, Abraham Oduro, Esther Oguk, Lucas Otieno, Walter Otieno, Seth Owusu-Agyei, Janet Oyieko, Jean-Yves Pirçon, Nicolas Praet, François Roman, Lode Schuerman, Valentine Sing’oei, Mathilda Tivura

**Affiliations:** Janssen Pharmaceutica NV, Beerse, Belgium

**Keywords:** Malaria, *Plasmodium falciparum*, Adverse event, Meningitis, Mortality

## Abstract

**Background:**

The lack of background disease incidence rates in sub-Saharan countries where the RTS,S/AS01_E_ malaria vaccine is being implemented may hamper the assessment of vaccine safety and effectiveness. This study aimed to document baseline incidence rates of meningitis, malaria, mortality, and other health outcomes prior to vaccine introduction through the Malaria Vaccine Implementation Programme.

**Methods:**

An ongoing disease surveillance study is combining prospective cohort event monitoring and hospital-based disease surveillance in three study sites in Ghana and Kenya. An interim analysis was performed on the prospective cohort in which children were enrolled in two age-groups (the 5 to 17 months or 6 to 12 weeks age-group), capturing data in the framework of routine medical practice before the introduction of the malaria vaccine. Incidence and mortality rates were computed with 95% confidential intervals (CI) using an exact method for a Poisson variable.

**Results:**

This analysis includes 14,329 children; 7248 (50.6%) in the 6 to 12 weeks age-group and 7081 (49.4%) in the 5 to 17 months age-group. In the 5 to 17 months age-group (where the malaria vaccine was planned to be subsequently rolled out) the meningitis, malaria, severe malaria and cerebral malaria incidences were 92 (95% CI 25–236), 47,824 (95% CI 45,411–50,333), 1919 (95% CI 1461–2476) and 33 (95% CI 1–181) per 100,000 person-years, respectively. The all-cause mortality was 969 (95% CI 699–1310) per 100,000 person-years.

**Conclusion:**

Incidence estimates of multiple health outcomes are being generated to allow before-after vaccine introduction comparisons that will further characterize the benefit-risk profile of the RTS,S/AS01_E_ vaccine.

*Trial registration:* clinicaltrials.gov NCT02374450.

**Supplementary Information:**

The online version contains supplementary material available at 10.1186/s12936-021-03670-w.

## Background

Despite remarkable progress in reducing the global burden of disease due to malaria, there were an estimated 228 million cases of malaria in 2018 and 405,000 malaria deaths globally, of which 380,000 occurred in the African region [1]. *Plasmodium falciparum* caused 99.7% of malaria cases, and children aged < 5 years were the most vulnerable age-group, accounting for 272,000 (67%) of global malaria deaths in 2018 [1]. Without additional tools such as vaccines, the World Health Organization (WHO) 2030 global targets of achieving at least a 90% reduction in malaria incidence and deaths compared to 2015 levels, may not be met [2].

RTS,S/AS01_E_ is a pre-erythrocytic *P. falciparum* malaria vaccine developed for routine immunization of infants and children living in malaria-endemic countries, and is the first complementary tool to be implemented under the Global Technical Strategy for Malaria, 2016–2030. RTS,S/AS01_E_ received a positive scientific opinion from the European Medicines Agency in 2015 [3]. Later that year the WHO recommended pilot implementation of routine RTS,S/AS01_E_ vaccination in sub-Saharan Africa (SSA), administering three doses to children aged 5 to 9 months in areas of moderate-to-high malaria transmission, with a fourth dose 15 to 18 months later [4]. This pilot implementation will provide more clarity on uncertainties related to programmatic feasibility, impact and safety of the vaccine in routine use [4].

In 2019, RTS,S/AS01_E_ was introduced in selected areas of Ghana, Kenya, and Malawi through national immunization programmes in the framework of the WHO-commissioned Malaria Vaccine Implementation Programme (MVIP) [5]. The MVIP is evaluating the vaccine’s public health impact in the context of routine use and will inform policy about its potential deployment on a broader scale. As part of this evaluation, GSK has committed to conduct a post-approval plan (PAP) in the pilot implementation areas to further assess vaccine safety, effectiveness and impact. Specific safety outcomes of interest included those identified during the Phase III clinical trial: an imbalance in meningitis and cerebral malaria cases between RTS,S/AS01_E_ and control vaccine groups in children that started vaccination at 5 to 17 months of age [6], and across both age groups a higher mortality in girls vaccinated with RTS,S/AS01_E_ versus girls vaccinated with control vaccines not explained by differences in risk factors, causes of death, or time to death. Detailed evaluation of these events and absence of a biological plausible explanation for a causal relationship to RTS,S/AS01_E_ suggest that these observations were likely chance findings, possibly due to a low mortality rate in girls who received control vaccines, or low rates of meningitis in the control group [6].

Limited or absent healthcare and disease surveillance infrastructure, and the lack of robust disease incidence data in SSA before RTS,S/AS01_**E**_ implementation [7], hamper the assessment of vaccine safety, effectiveness and impact. For this reason, the PAP design includes a before-after comparison in which data collected prior to (pre-vaccine introduction study; NCT02374450) and after (post-vaccine introduction study; NCT03855995) vaccine introduction are compared. Both studies are observational and follow a cohort study design to estimate incidence rates of meningitis, malaria, selected rare events (referred to here as adverse events of special interest [AESI]), other events that lead to hospitalization, and mortality in children aged less than 5 years. The present paper summarizes the interim results of this pre-vaccine introduction study that has been conducted so far in Ghana and Kenya in children not vaccinated with RTS,S/AS01_E_. The end of study results will be disclosed in a separate publication.

## Methods

### Study design and population

This pre-vaccine introduction study is a disease surveillance study with prospective cohort event monitoring including active surveillance of outpatient and inpatient visits by each enrolled study participant and scheduled home visits. In addition, hospital-based disease surveillance is organized in the entire study area for infants and young children not enrolled in the prospective cohort. The present interim results are limited to the analysis of data collected through the prospective cohort monitoring in which subjects are enrolled in two groups (either the 5 to 17 months or the 6 to 12 weeks age-groups) corresponding to the two age-groups evaluated in the RTS,S/AS01_E_ Phase III study [8]. Those age-groups differ in the way their active surveillance is organized i.e., 10 home visits are organized according to the diphtheria-tetanus-pertussis-hepatitis B-*Haemophilus influenzae* (DTP-HepB-Hib) vaccination schedule for the 6 to 12 weeks age-group, or from 5 months of age (according to a schedule mimicking administration of RTS,S/AS01_E_, and referred to hereafter as the ‘virtual primary vaccination schedule’) for the 5 to 17 months age-group (Additional file 1). The children, distributed equally between the two age-groups, were recruited into the prospective cohort from three study sites (Kintampo and Navrongo in central and northern Ghana, respectively; Kombewa in Kenya) from January 2016 until June 2018.

### Study objectives

The study objectives are to estimate the incidence of AESI (listed in Additional file 2), meningitis (aetiology-confirmed, probable, and clinically suspected meningitis), malaria (any, uncomplicated, severe, and cerebral malaria), other events leading to hospitalization, all-cause and malaria-attributable mortality rates (overall and by gender), and to describe causes of death (overall and by gender) in children prior to implementation of RTS,S/AS01_E_. Case definitions are described in Additional file 2. At-risk periods after DTP-HebB-Hib vaccination for the 6 to 12 weeks age group and virtual at-risk periods after a virtual vaccination mimicking RTS,S/AS01_E_ administration for 5 to 17 months age group were defined for each study outcome (Additional file 3).

### Study procedures

This study captures data generated in the framework of routine medical practice, which includes medical history, clinical diagnosis and results of locally conducted laboratory tests (referred to as first-line laboratory results). Training on pharmacovigilance and the diagnosis of AESI and meningitis was provided to all sites. Job aids (i.e., field guides with signs and symptoms of AESI and meningitis, and guidelines for diagnosis based on the case definition) were also provided, and laboratory testing capacities were enhanced to support case detection and ascertainment. For each suspected case of AESI and meningitis, a protocol-specified blood sample of 5 ml was sent to an external reference laboratory for testing (second-line laboratory). For meningitis and neurological AESI, when a lumbar puncture was conducted according to routine diagnostic practice, an aliquot of cerebrospinal fluid (CSF) was sent to the second-line laboratory if a sufficient quantity was available after first-line laboratory testing.

All cases of meningitis, cerebral malaria, and ambiguous cases of any other endpoint were reviewed by a panel of external experts (i.e., medical professionals with varied backgrounds who independently reviewed the cases). Cause of death was either established from the medical records when occurring at a healthcare facility, or through verbal autopsy for children who died in the community [9].

### Statistical analysis

The total enrolled cohort of the interim analysis included all children enrolled in the prospective cohort who, by 05 October 2018, had reached the study home visit number five which is planned approximately 6 months after a 3-dose schedule of DTP-HepB-Hib for the 6 to 12 weeks age-group (primary vaccination schedule) or after a virtual 3-dose primary vaccination schedule mimicking RTS,S/AS01_E_ administration for the 5 to 17 months age-group (virtual primary vaccination schedule). Study participants who reached home visit five after this date were excluded. The According-to-protocol (ATP) cohort included all children who met eligibility criteria (Additional file 4), complied with protocol-defined enrolment procedures, and who had no elimination criteria during the study.

Incidence rates were computed overall and by study site, and mortality rates were computed overall, by gender, and by study site using the data collected during a follow-up period of approximately 6 months after the (virtual) primary vaccination schedule. A 95% confidential interval (CI) was computed using an exact method for a Poisson variable. For the events with an at-risk period (Additional file 3), the incidence rate was computed by dividing the number of children reporting at least one event within the at-risk period after each (virtual) vaccinated dose by the accumulated at-risk period after (virtual) vaccination.

AEs (other than AESI, meningitis or malaria) leading to hospitalization and cause of death were coded according to the MedDRA preferred terms. Preferred terms of other AEs leading to hospitalization were grouped into medically relevant categories for the calculation of the incidence rates (Additional file 5).

### Quality indicators of surveillance

To assess the operational conduct of the study, two quality surveillance indicators were monitored in health care facilities; abscess at the injection site during the 7-day period after each dose of the (virtual) primary vaccination schedule; and foot positional deformation, as defined in Additional file 6.

### Ethics and consent

The study was conducted in accordance with Good Clinical Practice and Good Pharmacoepidemiology Practices, all applicable subject privacy requirements and the guiding principles of the Declaration of Helsinki. The study protocol and associated documents were approved by relevant Ethical Review Boards (Additional file 7). Written or witnessed and thumb written informed consent was obtained from the parent/ legal representative of each study participant prior to enrolment.

Anonymized individual participant data and study documents can be requested for further research from www.clinicalstudydatarequest.com.

## Results

### Study participants

**Fig. 1:**
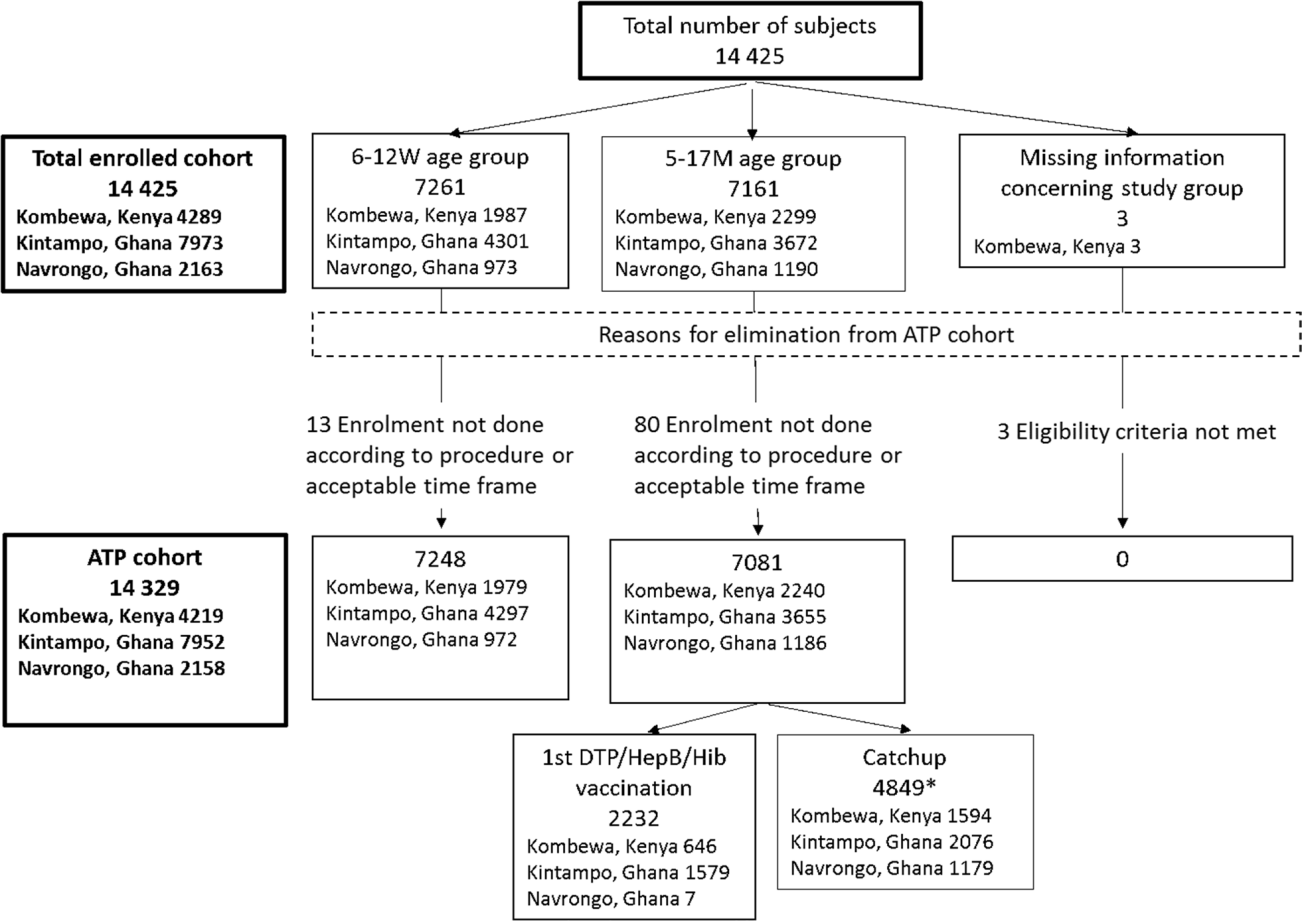
CONSORT diagram. KE, Kenya; GH, Ghana; ATP, According to protocol; M, Months; W, Weeks

A total of 14,425 children were enrolled in the prospective cohort, among which 14,329 were part of the ATP analysis: 4219 (29.4%) in Kombewa (Kenya), 7952 (55.5%) in Kintampo (central Ghana), and 2158 (15.1%) in Navrongo (northern Ghana) (Fig. 1). There were 7248 (50.6%) participants in the 6 to 12 weeks age-group and 7081 (49.4%) in the 5 to 17 months age-group (Fig. 1, Table 1).

**Table 1:** Number of study participants by study site and by study group

Study site	Study group	Number of participants (%) per study site
Kombewa, Kenya N = 4219	6 to 12 weeks	1979 (46.9)
	5 to 17 months	2240 (53.1)
Kintampo, Ghana N = 7952	6 to 12 weeks	4297 (54.0)
	5 to 17 months	3655 (46.0)
Navrongo, Ghana N = 2158	6 to 12 weeks	972 (45.0)
	5 to 17 months	1186 (55.0)

According-to-protocol cohort

N, total number of study participants by study site

In the ATP cohort, the mean age of study participants at enrolment was 1.7 months (standard deviation [SD] 0.3) in the 6 to 12 weeks age-group and 7.7 months (SD 5.1) in the 5 to 17 months age-group (Table 2). Males and females were equally distributed in both age-groups. Overall, 9745 (68.0%) study participants resided in rural areas and 12,958 (90.4%) lived less than 5 km from a healthcare facility. Details per study site are provided in Additional file 8.

**Table 2:** Demographic characteristics at study entry by study group

	6 to 12 weeks age-group (N = 7248)	5 to 17 months age-group (N = 7081)	Overall (N = 14,329)
Age at informed consent (months)			
Mean ± SD	1.7 ± 0.3	7.7 ± 5.1	4.7 ± 4.7
Range	0.4–3.0	0.4–18.0	0.4–18.0
Gender, n (%)			
Female	3585 (49.5)	3484 (49.2)	7069 (49.3)
Male	3663 (50.5)	3597 (50.8)	7260 (50.7)
Neighbourhood of residence, n (%)			
Urban	1320 (18.2)	920 (13.0)	2240 (15.6)
Semi-rural	1225 (16.9)	1119 (15.8)	2344 (16.4)
Rural	4703 (64.9)	5042 (71.2)	9745 (68.0)
Distance to health care facility, n (%)			
< 5 km	6588 (90.9)	6370 (90.0)	12,958 (90.4)
5– < 10 km	543 (7.5)	496 (7.0)	1039 (7.3)
10– < 20 km	84 (1.2)	149 (2.1)	233 (1.6)
20– < 30 km	17 (0.2)	42 (0.6)	59 (0.4)
> 30 km	16 (0.2)	24 (0.3)	40 (0.3)
Closest health care facility, n (%)			
Primary health care facility	5135 (70.9)	5165 (72.9)	10,300 (71.9)
Hospital	2113 (29.2)	1916 (27.1)	4029 (28.1)

According-to-protocol cohort

N, total number of study participants by study site; n, number of study participants in each category; SD: standard deviation

A total of 745 participants (5.2%) were hospitalized. Among the 5464 (38.1%) participants who had at least one outpatient visit, 32 (0.59%) were referred to hospital. The percentage of participants diagnosed for each endpoint is provided in Table 3.

**Table 3:** Percentage of study participants with final diagnoses for the main interim analysis endpoints

6 to 12 weeks	Kombewa, Kenya			Kintampo, Ghana			Navrongo, Ghana			Overall		
	N = 1979			N = 4297			N = 972			N = 7248		
	e	n	%	e	n	%	e	n	%	e	n	%
AESI	0	0	0.0	0	0	0.0	0	0	0.0	0	0	0.0
Meningitis	0	0	0.0	0	0	0.0	0	0	0.0	0	0	0.0
Malaria	479	375	19.0	898	685	15.9	48	43	4.4	1425	1103	15.2
Other AE^a^ leading to hospitalization	62	38	1.9	439	233	5.4	111	46	4.7	612	317	4.4
All-cause hospitalization	39	39	2.0	268	240	5.6	54	47	4.8	361	326	4.5

5 to 17 months	Kombewa, Kenya			Kintampo, Ghana			Navrongo, Ghana			Overall		
	N = 2240			N = 3655			N = 1186			N = 7081		
	e	n	%	e	n	%	e	n	%	e	n	%
AESI	1	1	0.04	1	1	0.0	0	0	0.0	2	2	0.0
Meningitis	3	3	0.1	1	1	0.0	0	0	0.0	4	4	0.1
Malaria	639	505	22.5	1363	938	22.7	127	118	10.0	2129	1561	22.0
Other AE^a^ leading to hospitalization	119	69	3.1	495	254	6.7	188	78	6.6	802	392	5.5
All-cause hospitalization	85	79	3.5	310	262	7.2	92	78	6.6	487	419	5.9

According-to-protocol cohort

AESI: adverse event of special interest; AE: adverse event; N: total number of study participants by study group or by study site; e, number of events reported; n, number of study participants in each category; %, percentage of study participants who reported at least one event

The AESI in this study refer to a list of 15 diseases: Acute disseminated encephalomyelitis, Encephalitis, Guillain-Barré syndrome, Generalized convulsive seizure, Hypotonic hypo-responsive episode, Intussusception, Hepatic insufficiency, Renal insufficiency, Juvenile chronic arthritis, Stevens Johnson syndrome and toxic epidermal necrolysis, Henoch Schonlein purpura, Kawasaki disease, Diabetes mellitus type 1, Thrombocytopenia, Anaphylaxis

^a^Adverse events other than AESI, malaria or meningitis

In the framework of the MVIP, the RTS,S/AS01_E_ primary vaccination schedule is currently administered to children aged 5 to 9 months. Therefore, the results of the ATP cohort presented below focus on the 5 to 17 months age-group. Results for the 6 to 12 weeks age-group are provided in Table 3 and Additional files 9–11.

### Incidence rates of meningitis

Five meningitis cases were initially suspected in the 5 to 17 months age-group, among which two cases were classified as aetiology-confirmed meningitis and two as clinically-suspected meningitis. The fifth case was subsequently diagnosed as encephalomyelitis (Table 4). None of the cases were fatal. CSF was available for three cases. There were two cases of aetiology-confirmed meningitis (incidence 46 (95% CI 6–167) per 100,000 person-years): one due to *Staphylococcus aureus,* and the other (case of co-morbidity with cerebral malaria; see below) incorrectly classified as meningitis due to *Parvovirus* before being downgraded to probable meningitis following further review by the expert panel after the interim analysis, and clarification of laboratory testing results. The incidence rate of aetiology-confirmed, probable and/or clinically suspected meningitis was 92 (95% CI 25–236) per 100,000 person-years.

**Table 4:** Incidence rate per 100,000 person-years of meningitis cases

	Incidence rate per 100,000 person-years		
	n	Person-years	Value (95% CI)
Kombewa, Kenya (N = 2111)			
Aetiology-confirmed meningitis	1	1249	80 (2, 446)
Probable meningitis	0	1249	0 (0, 295)
Clinically suspected meningitis	2	1249	160 (19, 579)
Aetiology-confirmed, probable, and/or clinically suspected meningitis	3	1248	240 (50, 703)
Suspected meningitis ruled out afterward	1	1249	80 (2, 446)
Kintampo, Ghana (N = 3603)			
Aetiology-confirmed meningitis^a^	1	2330	43 (1, 239)
Probable meningitis	0	2330	0 (0, 158)
Clinically suspected meningitis	0	2330	0 (0, 158)
Aetiology-confirmed, probable, and/or clinically suspected meningitis	1	2330	43 (1, 239)
Suspected meningitis ruled out afterward	0	2330	0 (0, 158)
Navrongo, Ghana (N = 1183)			
Aetiology-confirmed meningitis	0	754	0 (0, 490)
Probable meningitis	0	754	0 (0, 490)
Clinically suspected meningitis	0	754	0 (0, 490)
Aetiology-confirmed, probable, and/or clinically suspected meningitis	0	754	0 (0, 490)
Suspected meningitis ruled out afterward	0	754	0 (0, 490)
Overall (N = 6897)			
Aetiology-confirmed meningitis^a^	2	4332	46 (6, 167)
Probable meningitis	0	4332	0 (0, 85)
Clinically suspected meningitis	2	4332	46 (6, 167)
Aetiology-confirmed, probable, and/or clinically suspected meningitis	4	4331	92 (25, 236)
Suspected meningitis ruled out afterward	1	4332	23 (1, 129)

According-to-protocol cohort, 5 to 17 months age-group

N, number of study participants at risk during a follow-up period of approximately 6 months after each dose of the virtual primary vaccination schedule censored at the next dose; n, number of cases reported during that follow-up period; CI: confidence interval; M: Months

^a^One case of aetiology-confirmed meningitis has been downgraded to probable meningitis after the interim analysis (see text for details)

### Incidence rates of malaria

A total of 1561 participants (22%) experienced at least one episode of malaria (2129 episodes confirmed by rapid diagnostic test and/or microscopy) during an outpatient visit or a hospitalization. Uncomplicated malaria was diagnosed in 1512 children, which corresponds to 97% of the total number of malaria events (2059 out of 2129 episodes). The remaining 3% (70 episodes in 66 children) were severe and included two cases of cerebral malaria (fatal for one child, while the other child diagnosed with cerebral malaria and meningitis, recovered fully). *Plasmodium falciparum* was identified in 1747 cases (82%).

The malaria incidence rate was 47,824 (95% CI 45,411–50,333) per 100,000 person-years (Table 5). The incidence rates of uncomplicated and severe malaria were 45,905 (95% CI 43,541–48,364) and 1919 (95% CI 1461–2476) per 100,000 person-years, respectively. The cerebral malaria incidence rate was 33 (95% CI 1–181) per 100,000 person-years.

**Table 5:** Incidence rates per 100,000 person-years of malaria cases

	Kombewa, Kenya			Kintampo, Ghana			Navrongo, Ghana			Overall		
	N = 1743			N = 3488			N = 1143			N = 6374		
	n	PY	Value (95% CI)	n	PY	Value (95% CI)	n	PY	Value (95% CI)	n	PY	Value (95% CI)
Malaria												
Any	381	810	47,048 (42,442, 52,018)	1010	1714	58,928 (55,350, 62,678)	79	550	14,364 (11,372, 17,902)	1470	3074	47,824 (45,411, 50,333)
Uncomplicated	364	810	44,949 (40,449, 49,812)	972	1714	56,711 (53,202, 60,392)	75	550	13,637 (10,726, 17,094)	1411	3074	45,905 (43,541, 48,364)
Severe malaria	17	810	2099 (1223, 3361)	38	1714	2217 (1569, 3043)	4	550	727 (198, 1862)	59	3074	1919 (1461, 2476)
Cerebral malaria	0	810	0 (0, 456)	1	1714	58 (1, 325)	0	550	0 (0, 671)	1	3074	33 (1, 181)
*P. falciparum*												
Uncomplicated	277	810	34,205 (30,296, 38,480)	813	1714	47,434 (44,229, 50,810)	74	550	13,455 (10,565, 16,891)	1164	3074	37,869 (35,725, 40,109)
Severe	15	810	1852 (1037, 3055)	38	1714	2217 (1569, 3043)	4	550	727 (198, 1862)	57	3074	1854 (1405, 2403)

According-to-protocol cohort, 5 to 17 months age-group N, Number of study participants at risk during a follow-up period of approximately 6 months after the 3rd virtual vaccine dose; n, number of cases reported during that follow-up period; PY: person-years; CI: confidence intervals

Malaria cases were confirmed by rapid diagnostic test and/or microscopy

### Mortality rate

In total, 56 deaths (26 girls and 30 boys) were reported due to pneumonia (12 cases), malaria/*P. falciparum* infection (11 cases including one case of cerebral malaria), malnutrition (five cases), dehydration (four cases), gastroenteritis and thermal burn (three cases each), two cases each of asphyxia and herbal toxicity, one case each of acute chest syndrome, AIDS-related complication, anemia, bronchiolitis, HIV infection, infection, acute otitis media, pulmonary tuberculosis, renal insufficiency, sepsis, skin infection, sudden infant death syndrome, and two cases of death (cause unspecified). Approximately 29% of deaths occurred during hospitalization. The all-cause mortality rate per 100,000 person-years was 969 (95% CI 699–1310), with similar rates in girls (940, 95% CI 574–1451) and boys (998, 95% CI 626–1511) (Table 6). The rate of malaria-attributable deaths was 231 (95% CI 111–424) per 100,000 person-years (Table 6).

**Table 6:** All-cause mortality rate per 100,000 person-years by gender

	Kombewa, Kenya			Kintampo, Ghana			Navrongo, Ghana			Overall		
	n	PY	Value (95% CI)	n	PY	Value (95% CI)	n	PY	Value (95% CI)	n	PY	Value (95% CI)
Female	N = 1018			N = 1786			N = 584			N = 3388		
All causes	7	600	1166 (469, 2402)	12	1154	1040 (537, 1817)	1	374	267 (7, 1488)	20	2129	940 (574, 1451)
AESI	0	600	0 (0, 614)	0	1154	0 (0, 320)	0	374	0 (0, 985)	0	2129	0 (0, 173)
Meningitis	0	600	0 (0, 614)	0	1154	0 (0, 320)	0	374	0 (0, 985)	0	2129	0 (0, 173)
Malaria	0	600	0 (0, 614)	6	1154	520 (191, 1132)	0	374	0 (0, 985)	6	2129	282 (103, 614)
*P. falciparum*	0	600	0 (0, 614)	1	1154	87 (2, 483)	0	374	0 (0, 985)	1	2129	47 (1, 262)
Other AEs	7	600	1166 (469, 2402)	6	1154	520 (191, 1132)	1	374	267 (7, 1488)	14	2129	658 (360, 1104)
Male	N = 1093			N = 1817			N = 599			N = 3509		
All causes	14	649	2157 (1179, 3620)	7	1176	595 (239, 1227)	1	379	264 (7, 1469)	22	2204	998 (626, 1511)
AESI	0	649	0 (0, 568)	0	1176	0 (0, 314)	0	379	0 (0, 973)	0	2204	0 (0, 167)
Meningitis	0	649	0 (0, 568)	0	1176	0 (0, 314)	0	379	0 (0, 973)	0	2204	0 (0, 167)
Malaria	3	649	462 (95, 1351)	1	1176	85 (2, 474)	0	379	0 (0, 973)	4	2204	182 (49, 465)
*P. falciparum*	2	649	308 (37, 1113)	1	1176	85 (2, 474)	0	379	0 (0, 973)	3	2204	136 (28, 398)
Other AEs	11	649	1695 (846, 3033)	6	1176	510 (187, 1111)	1	379	264 (7, 1469)	18	2204	817 (484, 1291)
Total	N = 2111			N = 3603			N = 1183			N = 6897		
All causes	21	1249	1681 (1041, 2569)	19	2330	816 (491, 1274)	2	754	265 (32, 959)	42	4332	969 (699, 1310)
AESI	0	1249	0 (0, 295)	0	2330	0 (0, 158)	0	754	0 (0, 490)	0	4332	0 (0, 85)
Meningitis	0	1249	0 (0, 295)	0	2330	0 (0, 158)	0	754	0 (0, 490)	0	4332	0 (0, 85)
Malaria	3	1249	240 (50, 702)	7	2330	300 (121, 619)	0	754	0 (0, 490)	10	4332	231 (111, 424)
*P. falciparum*	2	1249	160 (19, 578)	2	2330	86 (10, 310)	0	754	0 (0, 490)	4	4332	92 (25, 236)
Other AEs	18	1249	1441 (854, 2277)	12	2330	515 (266, 900)	2	754	265 (32, 959)	32	4332	739 (505, 1043)

According-to-protocol cohort, 5 to 17 months age-group N, Number of study participants at risk during a follow-up period of approximately 6 months after each dose of the virtual primary vaccination schedule censored at the next dose; n, number of cases reported during that follow-up period

CI: confidence interval; PY: person-years; AE: adverse event; AESI: adverse event of special interest

### Incidence rates of AESI

Two AESI were diagnosed in participants in the 5 to 17 months age-group, one case of intussusception (144 per 100,000 person-years; 95% CI 4–800), and one case of encephalomyelitis occurring outside the at-risk period of 6 weeks (no incidence rate calculated). Both AESI led to hospitalization, neither was fatal.

### Other AEs leading to hospitalization

In the 5 to 17 months age-group, 392 study participants (5.5%) experienced 802 other events (i.e., other than meningitis, malaria or AESI) that led to their hospitalization (Table 3). The most frequently reported other events were anaemia, gastroenteritis, and lower respiratory tract infection (Table 7 and Additional file 12).

**Table 7:** Incidence rate per 100,000 person-year of other adverse events

Overall (N = 6897)	Incidence rate per 100,000 person-years			
	n	Person-years	Value (95% CI)	
Anemia	69	1490	4632	(3604, 5862)
Bacterial Infection	1	1492	67	(2, 373)
Burns	2	1492	134	(16, 484)
Conjunctivitis	1	1492	67	(2, 373)
Gastroenteritis	41	1491	2751	(1974, 3732)
Helminthic infection	2	1492	134	(16, 484)
Lower respiratory tract infection	38	1491	2549	(1804, 3499)
Malnutrition	3	1492	201	(41, 588)
Sepsis	22	1491	1475	(925, 2234)
Skin Infection	3	1492	201	(41, 588)
Upper respiratory tract infection	19	1492	1274	(767, 1989)
Urinary tract Infection	0	1492	0	(0, 247)

According-to-protocol cohort, 5 to 17 months age-group. Other adverse events, AE other than AESI, meningitis or malaria that was diagnosed during hospitalization.

Preferred terms of other AEs leading to hospitalization were grouped into medically relevant categories (refer to Additional file 5 for more details) for the calculation on the incidence rates. N, Number of study participants at risk within 30 days after each dose of the virtual primary vaccination schedule censored at the next dose; n, number of cases reported during that follow-up period

CI: confidence interval; M: Months; ATP: According-to-protocol cohort

### Quality indicators of surveillance

In the 6 to 12 weeks age-group, there were five cases of abscess at the injection site after a total of 21,149 DTP-HepB-Hib doses. No cases were reported in the 5 to 17 months age-group. No foot positional deformations were reported in either group.

## Discussion

In the context of the MVIP, the RTS,S/AS01_E_ malaria vaccine has recently been included in the Expanded Programmes on Immunization (EPI) of selected pilot implementation areas of Ghana, Kenya and Malawi. As RTS,S/AS01_E_ has not been introduced as part of EPI anywhere else in the world, no post-authorization surveillance data has been generated so far. The MVIP includes an important vaccine evaluation component in which GSK is conducting a comprehensive PAP in collaboration with African scientific partners. Limited background data on the incidence of diseases of interest in the framework of this PAP are available in the SSA context. The present study enrolled children not vaccinated with RTS,S/AS01_E_ in three study sites of moderate-to-high malaria transmission in Ghana and Kenya, countries where RTS,S/AS01_E_ is being administered in selected areas since 2019. The study was designed to document baseline incidence data prior to RTS,S/AS01_E_ implementation to allow a before-after vaccine introduction comparison, and more broadly to help interpretation of pharmacovigilance monitoring data after vaccine implementation. Preliminary study results pertaining to the 5 to 17 months age-group are discussed here.

Overall, the mortality rate per 100,000 person-years observed in the 5 to 17 months age-group was 969 (95% CI 699–1310) ranging from 265 (95% CI 32–959) to 1681 (95% CI 1041–2569) depending on the study site. Even though a formal comparison is not possible as age-groups and study period differ, those figures are not inconsistent with the INDEPTH Network mortality estimates for the period 2006 to 2012, ranging from 820 per 100,000 person-years in children aged 1 to 4 years in Navrongo, Ghana, to 7420 per 100,000 person-years in children aged 1 to 11 months in Kisumu, Kenya [10]. Overall, no marked difference in mortality rate between genders was observed.

Across the study sites, the incidence rate of meningitis (including aetiology-confirmed, probable and clinically suspected meningitis) was 92 (95% CI 25–236) per 100,000 person-years in the 5 to 17 months age-group. Of note, antibiotic therapy was initiated one to two days before CSF sample collection for two cases, which may have affected the CSF testing results and the final case classification. Though available data on meningitis incidence in SSA are scarce and clear case definitions are often lacking, this figure is in the range of incidence rates published for Burkina Faso (between 42 and 101 per 100,000 person-years in children aged less than 5 years for the 2011 to 2015 period) [11].

The incidence of malaria in the 5 to 17 months age-group was similar between Kintampo, Ghana and Kombewa, Kenya (as could be expected based on their similar malaria transmission intensity reported previously [12]) but fourfold lower in Navrongo, Ghana, emphasizing the need for generating local data when assessing vaccine effects. Incidence rates were estimated according to the level of disease severity, i.e., uncomplicated or severe (including cerebral) malaria, and following strict WHO case definitions. As clinical manifestations of malaria are highly age-dependent, especially in young children, final study results covering a longer follow-up period of enrolled children will provide key data that will be compared with post-approval data to assess both short and long-term vaccine effectiveness and impact.

Among the list of 15 defined AESI, only one case of intussusception (IS) occurred during the pre-defined risk period (14 days in the case of IS). It is not surprising to observe few AESIs at this stage given that these health outcomes are rare and that the current interim study results include a short subject follow-up period (6 months after the virtual primary vaccination schedule). The estimated incidence rate of IS was 144 (95% CI 4–800) per 100,000 person-years. Although the precision around this estimate is low, it is in the range of previously published data (mean incidence rate of 74 [range 9–328] per 100,000 person-years in children less than 1 year old between 1978 and 2012) [13].

The operational conduct of the study was assessed by two quality surveillance indicators. The rate of abscess at injection site observed for the 6 to 12 weeks age-group (24 events per 100,000 DTP-HepB-Hib doses) is within the expected range for this vaccine (22 to 108 cases per 100,000 doses) [14]. The absence of cases in the 5 to 17 months age-group was not unexpected as that assessment did not follow a real vaccine injection (i.e. virtual vaccination schedule mimicking RTS,S/AS01_E_ administration). No foot positional deformations were reported in either group. As the study participants were around 6 weeks of age (corresponding to the 1st DTP-HepB-Hib vaccination dose) or older at time of enrolment, the choice of a congenital anomaly was not appropriate.

This study also generated incidence rates of more frequent conditions affecting children in SSA, such as anemia, gastroenteritis, respiratory infections, and sepsis, for which incidence estimates are often lacking [15, 16]. To a broader extent, the incidence rates reported here will support international efforts to estimate the global burden of diseases, especially in the SSA region.

This study has been designed to detect conditions that require access to specific diagnostic tools and expertise that is not systematically available in SSA. For this reason, multiple tools have been developed and put in place to enhance case detection/ascertainment, e.g., medical trainings, job aids, telemedicine support, second-line laboratory testing of CSF/serum samples, local laboratory support, home visits, and consultation of an external expert panel. Among other aspects, those tools have been pivotal in enhancing the routine practice of CSF collection to appropriately investigate cases of suspected meningitis, and to differentiate these with cerebral malaria, combining both first and second line laboratory results and medical judgement to reach the final diagnosis. Despite the strong capacity building and subject follow-up components of this study, data collection relies on routine practice and optimal case detection and ascertainment may not have been reached due to the complex setting. Additionally, shortages of medical supplies, limited radiological and laboratory investigational capacities and/or reduced availability of medical personnel during strikes and elections are events that may have influenced case detection and ascertainment. However, this should not affect future vaccine safety and effectiveness assessment as the post-introduction study is based on the same design and is conducted in the same conditions.

## Conclusion

These preliminary results are key for several reasons: (1) they are the backbone of the RTS,S/AS01_E_ PAP, generating background incidence figures around which the before-after vaccine introduction comparison will be articulated; (2) they provide baseline figures to support pharmacovigilance monitoring of the vaccine; (3) they demonstrate the technical and operational feasibility of conducting such a large scale prospective cohort study with limited pre-existing health care and surveillance infrastructures; (4) beyond the RTS,S/AS01_E_ PAP, they provide incidence rate estimates of health outcomes for which only simulated/modelled figures are usually available. At the end of both pre- and post-vaccine introduction studies, all subjects enrolled in the prospective cohorts will have been followed up until 2 years after the fourth scheduled vaccination dose. This time-period will cover risks periods after each vaccine dose administration, as well as assessing vaccine effectiveness and impact after the primary vaccination schedule and the booster dose.

**Fig. 2:**
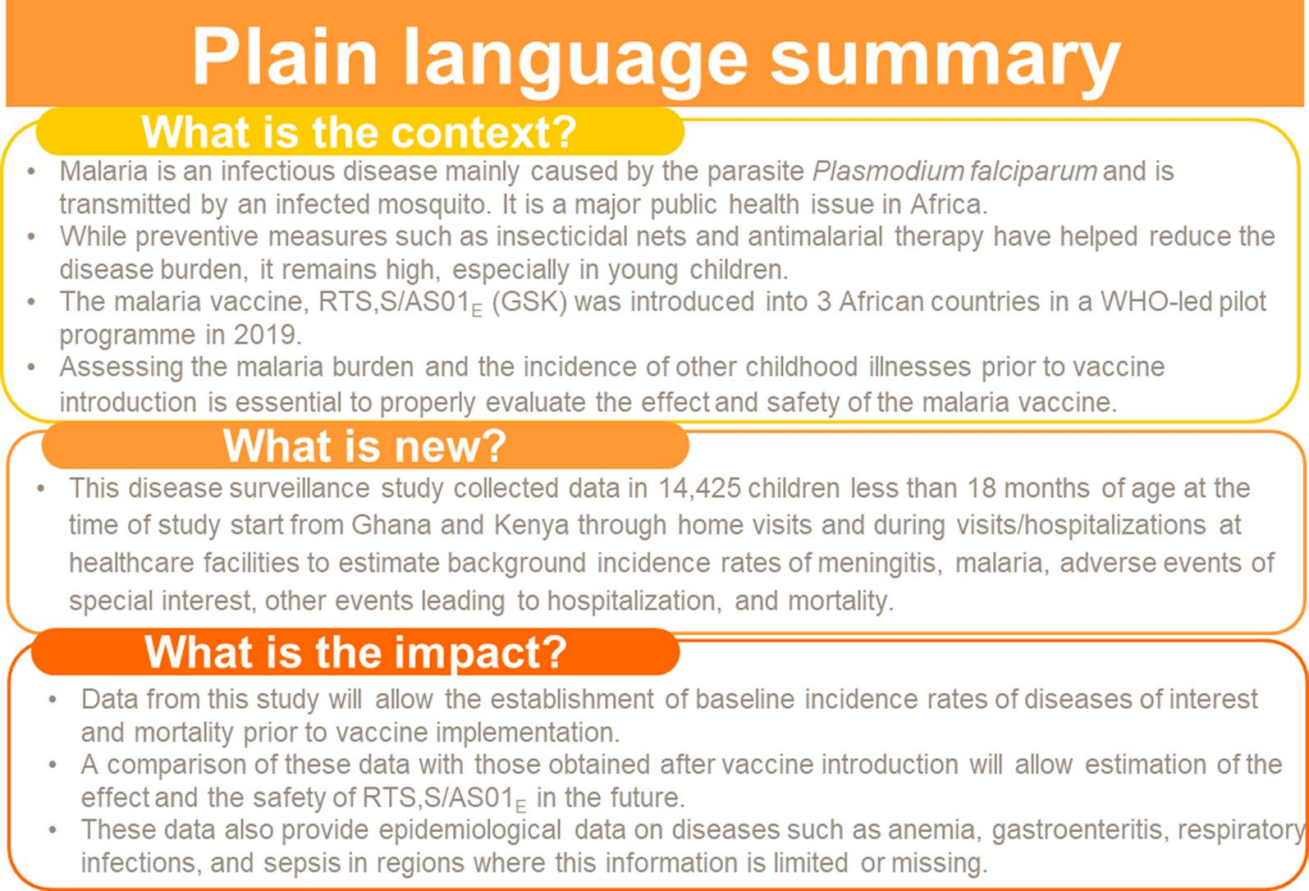
Plain Language Summary

A plain language summary of the study is provided in Fig. 2.

## Supplementary Information

**Additional file 1.** Study age groups and home visits

**Additional file 2.** Case definitions

**Additional file 3.** Pre-defined at-risk periods after the (virtual) primary vaccination schedule

**Additional file 4.** Eligibility criteria

**Additional file 5.** Grouping of other adverse events leading to hospitalization

**Additional file 6.** Quality surveillance indicators

**Additional file 7.** List of ethical review boards

**Additional file 8.** Demographic characteristics at study entry by study group and study site, According-to-protocol cohort

**Additional file 9.** Incidence rate per 100,000 person-years of malaria cases. According-to-protocol cohort, 6 to 12 weeks age-group

**Additional file 10.** All-cause mortality rate per 100,000 person-years by gender. According-to protocol-cohort, 6 to 12 weeks age-group

**Additional file 11.** Incidence rate per 100,000 person-years of other adverse events leading to hospitalization. ATP, 6 to 12 weeks age-group

**Additional file 12.** Incidence rate of other adverse events leading to hospitalization, by study site. ATP, 5 to 17 months age-group

## Data Availability

GSK makes available anonymized individual participant data and associated documents from interventional clinical studies which evaluate medicines, upon approval of proposals submitted to www.clinicalstudydatarequest.com. To access data for other types of GSK sponsored research, for study documents without patient-level data and for clinical studies not listed, please submit an enquiry via the website.

## Abbreviations

AE: Adverse event
AESI: Adverse event of special interest
ATP: According-to-protocol
CI: Confidence interval
CSF: Cerebrospinal fluid
DTP-HepB-Hib: Diphtheria-tetanus-pertussis-hepatitis B-*Haemophilus influenzae*
EPI: Expanded programme on immunization
IS: Intussusception
M: Months
MVIP: Malaria vaccine implementation programme
PAP: Post-approval plan
SSA: Sub-Saharan Africa
W: Weeks
