# Correction to: Baseline incidence of meningitis, malaria, mortality and other health outcomes in infants and young sub-Saharan African children prior to the introduction of the RTS,S/AS01_E_ malaria vaccine

**DOI:** 10.1186/s12936-021-03751-w

**Published:** 2021-06-07

**Authors:** Valérie Haine, The RTS,S Epidemiology EPI-MAL-002 Study Group

**Affiliations:** grid.425090.a GSK, Wavre, Belgium

## Correction to: Malar J (2021) 20:197 10.1186/s12936-021-03670-w

Following publication of the original article [1], the authors flagged that the article had published with incorrect affiliation details for the corresponding author; the article had published with the affiliation ‘Janssen Pharmaceutica NV, Beerse, Belgium’, while the correct affiliation is ‘‘GSK, Wavre, Belgium’.

In addition, incomplete affiliation details had been provided for Nicolas Praet in the Acknowledgements of the article; the complete affiliation is as follows: GSK, Wavre, Belgium; current affiliation: Janssen Pharmaceutica NV, Beerse, Belgium.

The original article has since been corrected and the correct affiliation details may be found in this correction.

The publisher apologizes for any inconvenience caused.
