# Supplementary material for: Baseline incidence of meningitis, malaria, mortality and other health outcomes in infants and young sub-Saharan African children prior to the introduction of the RTS,S/AS01E malaria vaccine
Source: Malar J. 2021 Apr 26;20:197. doi: 10.1186/s12936-021-03670-w (PMC8073890; doi:10.1186/s12936-021-03670-w)
Supplement: Supplementary file 1 — Additional file 1. Study age groups and home visits [file 12936_2021_3670_MOESM1_ESM.docx]

Additional file 1 Study age groups and home visits

The 6 to 12 weeks age group comprised children identified at first administration of routine DTP-HepB-Hib vaccine usually administered at 6 weeks of age. For this group, study home visits were conducted approximately 1 week after administration of each DTP-HepB-Hib vaccine (V1, V2, V3) then 6 weeks (V4), 6 months (V5) and 18 months after administration of the last dose of DTP-HepB-Hib vaccine (V6) followed by visits approximately 5 weeks (V7), 12 months (V8) and 24 months (V9) after V6.

The 5 to 17 months age group includes a group of children identified at first administration of DTP-HepB-Hib vaccine and a group of children aged 5 to 17 months identified at first encounter with the study staff, such as during home visit or hospitalization. The first study home visit (V1) was to be at approximately 6 months of age for children identified at first administration of DTP-HepB-Hib vaccine or within one week of the first encounter with study staff for children aged 5 to <18 months, The following study home visits were to be scheduled in a similar way as for the 6 to 12 weeks age group, with one month interval between the first 3 visits (mimicking visits done 1 week after each dose of the RTS,S primary vaccination schedule) and a follow-up (V4 to V9) continuing for up to 44 months (24 months after V6), corresponding to the follow-up period of children enrolled in the post-introduction study (24 months after the fourth dose of RTS,S/AS01_E_).

A last study home visit (V10) will be conducted at study conclusion for all study participants (at 5 years of age or at time of visit 9 of the last participant enrolled per site, whichever comes first).
