# Supplementary material for: Baseline incidence of meningitis, malaria, mortality and other health outcomes in infants and young sub-Saharan African children prior to the introduction of the RTS,S/AS01E malaria vaccine
Source: Malar J. 2021 Apr 26;20:197. doi: 10.1186/s12936-021-03670-w (PMC8073890; doi:10.1186/s12936-021-03670-w)
Supplement: Supplementary file 2 — Additional file 2. Case definitions [file 12936_2021_3670_MOESM2_ESM.docx]

Additional file 2 Case definitions

***Adverse events of special interest (AESI)***

The AESI in this study refer to a list of 15 diseases:

- Acute disseminated encephalomyelitis
- Encephalitis
- Guillain-Barré syndrome
- Generalized convulsive seizure
- Hypotonic hypo-responsive episode
- Intussusception
- Hepatic insufficiency
- Renal insufficiency
- Juvenile chronic arthritis
- Stevens Johnson syndrome and toxic epidermal necrolysis
- Henoch Schonlein purpura
- Kawasaki disease
- Diabetes mellitus type 1
- Thrombocytopenia
- Anaphylaxis.

These AESI, developed in collaboration with a group of pediatricians working in SSA, were selected because they have historically been associated with other vaccines than RTS,S/AS01_E_, or may be hypothetically associated with RTS,S/AS01_E_ because this vaccine has components which are new compared to current widely used vaccines.

When available, the Brighton Collaboration Working Groups case definitions were used.

***Meningitis***

Case definitions were adapted from the WHO 2003.[[1](#_ENREF_1)]

A suspected meningitis case based on clinical symptoms and/or signs is defined as a child with sudden onset of fever (>38.0°C rectal or 37.5°C axillary) and one or more of the following signs: neck stiffness, altered consciousness with no other alternative diagnosis, or other meningeal sign such as bulging fontanelle in children under 1 year of age.

Lumbar puncture was to be performed according to routine medical practice and national recommendations for examination of cerebrospinal fluid (CSF).

After laboratory testing (done by the study site and by the referral laboratory) and after external panel of experts’ review, final classification of meningitis cases was to be as follows:

- **Etiology-confirmed meningitis case**: a CSF sample was available and a known etiologic agent (bacterial or not) was identified.
- **Probable meningitis case**: a CSF sample was available, no etiologic agent was identified but there were some abnormalities in the CSF (such as turbid macroscopic aspect, positive Gram, positive antigen test, pleiocytosis, abnormal glucose or protein levels), or positive blood culture.
- **Clinically suspected meningitis case**: a CSF sample was available and all laboratory results were normal after second line laboratory results, or no CSF sample was available and no alternative diagnosis was evident.
- **No meningitis case:** no specific clinical symptoms and/or signs or laboratory results were fulfilled.

***Malaria***

Cases were defined according to the WHO.[[2](#_ENREF_2)]

Uncomplicated malaria: Plasmodium parasitemia > 0 detected by microscopy and/or rapid diagnostic test

AND

Presence of fever (temperature ≥ 37.5°C), as reported by the parent(s)/LAR(s) or recorded at the time of presentation

OR

Occurring in a child who was unwell and brought for treatment to a health care facility.

AND

Without signs of severity or evidence (clinical or laboratory) of vital organ dysfunction.

Severe falciparum malaria: *P. falciparum* parasitemia > 0 detected by microscopy and/or rapid diagnostic test

AND

One or more of the following, occurring in the absence of an identified alternative cause:

- Impaired consciousness: a Glasgow coma score <11 in children ≥2 years of age or a Blantyre coma score <3 in children <2 years of age;
- Prostration: generalized weakness, unable to sit, stand or walk without assistance;
- Multiple convulsions: more than two episodes within 24 h;
- Acidosis: a base deficit of >8 mEq/L or, if not available, a plasma bicarbonate level of < 15 mmol/L or venous plasma lactate ≥5 mmol/L. Severe acidosis manifested clinically as respiratory distress (rapid, deep, laboured breathing).
- Hypoglycemia: blood or plasma glucose <2.2 mmol/L (<40 mg/dL);
- Severe malarial anemia: hemoglobin concentration ≤5 g/dL or a hematocrit of ≤15% in children <12 years of age with a parasite count >10,000/µL;
- Renal impairment: plasma or serum creatinine >265 μmol/L (3 mg/dL) or blood urea >20 mmol/L;
- Jaundice: plasma or serum bilirubin >50 μmol/L (3 mg/dL) with a parasite count >100,000/µL;
- Pulmonary edema: radiologically confirmed or oxygen saturation <92% on room air with a respiratory rate >30/min, often with chest indrawing and crepitations on auscultation;
- Significant bleeding: including recurrent or prolonged bleeding from the nose, gums or venepuncture sites; hematemesis or melena;
- Shock: compensated shock was defined as capillary refill ≥3 s or temperature gradient on leg (mid to proximal limb), but no hypotension. Decompensated shock was defined as systolic blood pressure <70 mm Hg in children, with evidence of impaired perfusion (cool peripheries or prolonged capillary refill);
- Hyperparasitemia: *P. falciparum* parasitemia >10% (i.e. percentage of infected red blood cells >10%; corresponding to >500,000/µL).

Cerebral malaria: Severe *P. falciparum* malaria with impaired consciousness (Glasgow coma score <11 in children ≥2 years of age or Blantyre coma score <3 in children <2 years of age);

AND

If malaria with seizure: coma persisting for >30 min after the seizure.

Other treatable causes of coma were to be excluded before diagnosing cerebral malaria (e.g. hypoglycemia, bacterial meningitis).

References

1. WHO case definition, 2003. WHO Coordinated Invasive Bacterial Vaccine Preventable Diseases (IB-VPD) Surveillance Network: Tier 1 Meningitis Surveillance. <https://www.who.int/immunization/monitoring_surveillance/resources/IB-VPD_Case_Defs.pdf> Accessed 26 February 2019.

2. World Health Organization. Guidelines for the treatment of malaria. 3rd Edition. 2015. <http://www.who.int/malaria/publications/atoz/9789241549127/en/>. Accessed 26 February 2019.
