# Supplementary material for: Baseline incidence of meningitis, malaria, mortality and other health outcomes in infants and young sub-Saharan African children prior to the introduction of the RTS,S/AS01E malaria vaccine
Source: Malar J. 2021 Apr 26;20:197. doi: 10.1186/s12936-021-03670-w (PMC8073890; doi:10.1186/s12936-021-03670-w)
Supplement: Supplementary file 3 — Additional file 3. Pre-defined at-risk periods after the (virtual) primary vaccination schedule [file 12936_2021_3670_MOESM3_ESM.docx]

Additional file 3 Pre-defined at-risk periods after the (virtual) primary vaccination schedule*

| **Events** | **At-risk period** |
| --- | --- |
| Meningitis | 12 months** |
| Mortality | 12 months** |
| Cerebral malaria | 12 months** |
| Acute disseminated encephalomyelitis  Encephalitis | 6 weeks  6 weeks |
| Guillain Barré Syndrome | 3 months |
| Generalized convulsive seizure | 2 weeks |
| Hypotonic Hypo-responsive Episode | 2 weeks |
| Intussusception | 2 weeks |
| Hepatic insufficiency | 2 weeks |
| Renal insufficiency | 2 weeks |
| Juvenile Chronic Arthritis | 6 months |
| Stevens Johnson Syndrome, Toxic epidermal necrolysis | 6 weeks |
| Henoch Schonlein purpura | 6 months |
| Kawasaki disease | 6 weeks |
| Diabetes mellitus type 1 | 6 months |
| Thrombocytopenia | 6 weeks |
| Anaphylaxis  Other AEs leading to hospitalization | 2 weeks  30 days |

* including an at-risk period following each dose of the vaccine censored at the administration of the following dose

** restricted to the available follow-up period at the time of the interim analysis (approximately 6 months after the [virtual] primary vaccination schedule).
