# Supplementary material for: Baseline incidence of meningitis, malaria, mortality and other health outcomes in infants and young sub-Saharan African children prior to the introduction of the RTS,S/AS01E malaria vaccine
Source: Malar J. 2021 Apr 26;20:197. doi: 10.1186/s12936-021-03670-w (PMC8073890; doi:10.1186/s12936-021-03670-w)
Supplement: Supplementary file 4 — Additional file 4. Eligibility criteria [file 12936_2021_3670_MOESM4_ESM.docx]

Additional file 4 Eligibility criteria

***Inclusion criteria***

- Subjects’ whose parent(s)/legal representative, in the opinion of the investigator, can and will comply with the requirements of the protocol.
- Written informed consent provided by the parent(s)/legal representative.
- Subject living within the health and demographic surveillance system (HDSS) area.
- Children must be <18 months of age for enrolment in the prospective cohort monitoring through active surveillance

***Exclusion criteria***

- Child in care
