# Supplementary material for: Baseline incidence of meningitis, malaria, mortality and other health outcomes in infants and young sub-Saharan African children prior to the introduction of the RTS,S/AS01E malaria vaccine
Source: Malar J. 2021 Apr 26;20:197. doi: 10.1186/s12936-021-03670-w (PMC8073890; doi:10.1186/s12936-021-03670-w)
Supplement: Supplementary file 5 — Additional file 5. Grouping of other adverse events leading to hospitalization [file 12936_2021_3670_MOESM5_ESM.docx]

Additional file 5 Grouping of other adverse events leading to hospitalization

| **10 most frequent adverse event groups** | **MedDRA preferred term (version 21.1)** |
| --- | --- |
| Anemia | Anemia |
|  | Iron deficiency anemia |
|  | Sickle cell anemia |
| Gastroenteritis | Gastroenteritis |
|  | Shigella infection |
|  | Typhoid fever |
|  | Amoebic dysentery |
|  | Gastroenteritis bacterial |
|  | Gastroenteritis salmonella |
|  | Gastrointestinal viral infection |
| Lower respiratory tract infection | Pneumonia |
|  | Pneumonia bacterial |
|  | Pneumonia pneumococcal |
|  | Pneumonia aspiration |
|  | Lower respiratory tract infection |
|  | Bronchitis |
|  | Bronchiolitis |
| Sepsis | Sepsis |
|  | Salmonella sepsis |
|  | Staphylococcal sepsis |
| Upper respiratory tract infection | Upper respiratory tract infection |
|  | Otitis media |
|  | Otitis media acute |
|  | Nasopharyngitis |
| Skin infection | Staphylococcal impetigo |
|  | Skin infection |
|  | Dermatitis diaper |
|  | Pyoderma |
|  | Impetigo |
|  | Carbuncle |
|  | Cellulitis |
|  | Furuncle |
|  | Fungal skin infection |
|  | Subcutaneous abscess |
| Malnutrition | Malnutrition |
| Conjunctivitis | Conjunctivitis |
| Helminthic infection | Helminthic infection |
| Urinary tract infection | Urinary tract infection |
| Bacterial infection | Bacterial infection |
| Burn | Thermal burn |
|  | Burns second degree |

The adverse events other than meningitis, adverse events of special interest and malaria that led to hospitalization were coded according to the MedDRA preferred terms and pooled into medically relevant groups. The 10 most frequent groups are displayed here, and incidence rates are presented in tables 7 and S7.
