# Supplementary material for: Baseline incidence of meningitis, malaria, mortality and other health outcomes in infants and young sub-Saharan African children prior to the introduction of the RTS,S/AS01E malaria vaccine
Source: Malar J. 2021 Apr 26;20:197. doi: 10.1186/s12936-021-03670-w (PMC8073890; doi:10.1186/s12936-021-03670-w)
Supplement: Supplementary file 6 — Additional file 6. Quality surveillance indicators [file 12936_2021_3670_MOESM6_ESM.docx]

Additional file 6 Quality surveillance indicators

***Abscess at injection site***

The diagnosis and monitoring of abscess at the injection site was used as a positive control as this is an adverse event that is relatively frequently observed after routine vaccination in Africa and was defined as:

- A localized collection of material in subcutaneous tissue, fat, fascia or muscle at the site of immunization confirmed in spontaneous or surgical drainage of material from the mass or by presence of palpable fluctuance (defined as a wavelike motion on palpation due to liquid content). The abscess may be further classified as due to infectious etiology, a sterile abscess or not-determined. Abscesses of infectious etiology may be accompanied by fever/regional lymphadenopathy. Sterile abscesses are not accompanied by fever/regional lymphadenopathy.

***Foot positional deformation***

The diagnosis and monitoring of foot positional deformation as a birth defect was used as a negative control and was defined as:

- Metatarsus adductus characterized by medial deviation (adduction) of the forefoot while the hind-foot remains in a normal position, thus forming a "C" shape, or concavity of the medial aspect of the foot

OR

- Positional calcaneovalgus characterized by hyperdorsiflexion of the foot with the abduction of the forefoot, which often results in the forefoot resting on the anterior surface of the lower leg

OR

- Clubfoot characterized by the foot being excessively plantar flexed, with the forefoot swung medially and the sole facing inward
