# Supplementary material for: Baseline incidence of meningitis, malaria, mortality and other health outcomes in infants and young sub-Saharan African children prior to the introduction of the RTS,S/AS01E malaria vaccine
Source: Malar J. 2021 Apr 26;20:197. doi: 10.1186/s12936-021-03670-w (PMC8073890; doi:10.1186/s12936-021-03670-w)
Supplement: Supplementary file 7 — Additional file 7. List of ethical review boards [file 12936_2021_3670_MOESM7_ESM.docx]

Additional file 7 List of ethical review boards

- Kintampo, Ghana:
  - Kintampo Health Research Centre Institutional Ethics Committee
  - Ghana Health Service Ethical Review Committee
  - London School of Hygiene & Tropical Medicine Research Ethics Committee
- Kombewa, Kenya:
  - Division of Human Subjects Protection, Walter Reed Army Institute of Research
  - Scientific and Ethics Review Unit, Kenya Medical Research Institute
- Navrongo, Ghana:
  - Navrongo Health Research Institutional Review Board (NHRC IRB), Navrongo
  - Ghana Health Service Ethics Review Committee (GHSERC), Accra
- PATH Research Ethics Committee

References

1. **WHO case definition, 2003. WHO Coordinated Invasive Bacterial Vaccine Preventable Diseases (IB-VPD) Surveillance Network: Tier 1 Meningitis Surveillance.** [**https://www.who.int/immunization/monitoring_surveillance/resources/IB-VPD_Case_Defs.pdf**](https://www.who.int/immunization/monitoring_surveillance/resources/IB-VPD_Case_Defs.pdf) **Accessed 26 February 2019.**

2. **World Health Organization. Guidelines for the treatment of malaria. 3rd Edition. 2015.** [**http://www.who.int/malaria/publications/atoz/9789241549127/en/**](http://www.who.int/malaria/publications/atoz/9789241549127/en/)**. Accessed 26 February 2019.**
