# Supplementary material for: Baseline incidence of meningitis, malaria, mortality and other health outcomes in infants and young sub-Saharan African children prior to the introduction of the RTS,S/AS01E malaria vaccine
Source: Malar J. 2021 Apr 26;20:197. doi: 10.1186/s12936-021-03670-w (PMC8073890; doi:10.1186/s12936-021-03670-w)
Supplement: Supplementary file 8 — Additional file 8. Demographic characteristics at study entry by study group and study site, According-to-protocol cohort [file 12936_2021_3670_MOESM8_ESM.docx]

Additional file 8 Demographic characteristics at study entry by study group and study site, According-to-protocol cohort

|  | **6 to 12 weeks N=7428** | | | **5 to 17 months N=7081** | | |
| --- | --- | --- | --- | --- | --- | --- |
|  | **Kombewa, Kenya** | **Kintampo, Ghana** | **Navrongo, Ghana** | **Kombewa,**  **Kenya** | **Kintampo,**  **Ghana** | **Navrongo,**  **Ghana** |
| **Age at informed consent (months)** |  |  |  |  |  |  |
| Mean ± SD | 1.5 ± 0.2 | 1.8 ± 0.3 | 1.5 ± 0.2 | 7.5 ± 4.8 | 6.6 ± 5.1 | 11.6 ± 3.4 |
| Range | 0.4–3.0 | 0.6–2.9 | 0.8–2.9 | 1.31–18.0 | 0.4–17.9 | 1.5–17.8 |
| **Gender, n (%)** |  |  |  |  |  |  |
| Female | 965 (48.8) | 2110 (49.1) | 510 (52.5) | 1088 (48.6) | 1811 (49.6) | 585 (49.3) |
| Male | 1014 (51.2) | 2187 (50.9) | 462 (47.5) | 1152 (51.4) | 1844 (50.5) | 601 (50.7) |
| **Neighbourhood of residence, n (%)** |  |  |  |  |  |  |
| Urban | 32 (1.6) | 1160 (27.0) | 128 (13.2) | 26 (1.2) | 768 (21.0) | 126 (10.6) |
| Semi-rural | 330 (16.7) | 895 (20.8) | 0 (0) | 390 (17.4) | 729 (20.0) | 0 (0) |
| Rural | 1617 (81.7) | 2242 (52.2) | 844 (86.8) | 1824 (81.4) | 2158 (59.0) | 1060 (89.4) |
| **Distance to health care facility, n (%)** |  |  |  |  |  |  |
| <5 km | 1919 (97.0) | 3711 (86.4) | 958 (98.6) | 2030 (90.6) | 3174 (86.8) | 1166 (98.3) |
| 5 – <10 km | 59 (3.0) | 472 (11.0) | 12 (1.2) | 181 (8.1) | 298 (8.2) | 17 (1.4) |
| 10 – <20 km | 1 (0.1) | 82 (1.9) | 1 (0.1) | 25 (1.1) | 121 (3.3) | 3 (0.3) |
| 20 – <30 km | 0 (0) | 17 (0.4) | 0 (0) | 4 (0.2) | 38 (1.0) | 0 (0) |
| >30 km | 0 (0) | 15 (0.4) | 1 (0.1) | 0 (0) | 24 (0.7) | 0 (0) |
| **Closest health care facility, n (%)** |  |  |  |  |  |  |
| Primary health care facility | 1105 (55.8) | 3146 (73.2) | 884 (91.0) | 1375 (61.4) | 2669 (73.0) | 1121 (94.5) |
| Hospital | 874 (44.2) | 1151 (26.8) | 88 (9.1) | 865 (38.6) | 986 (27.0) | 65 (5.5) |

N, total number of study participants by study site; n, number of study participants in each category; SD, standard deviation.

References

1. **WHO case definition, 2003. WHO Coordinated Invasive Bacterial Vaccine Preventable Diseases (IB-VPD) Surveillance Network: Tier 1 Meningitis Surveillance.** [**https://www.who.int/immunization/monitoring_surveillance/resources/IB-VPD_Case_Defs.pdf**](https://www.who.int/immunization/monitoring_surveillance/resources/IB-VPD_Case_Defs.pdf) **Accessed 26 February 2019.**

2. **World Health Organization. Guidelines for the treatment of malaria. 3rd Edition. 2015.** [**http://www.who.int/malaria/publications/atoz/9789241549127/en/**](http://www.who.int/malaria/publications/atoz/9789241549127/en/)**. Accessed 26 February 2019.**
