# Supplementary material for: Baseline incidence of meningitis, malaria, mortality and other health outcomes in infants and young sub-Saharan African children prior to the introduction of the RTS,S/AS01E malaria vaccine
Source: Malar J. 2021 Apr 26;20:197. doi: 10.1186/s12936-021-03670-w (PMC8073890; doi:10.1186/s12936-021-03670-w)
Supplement: Supplementary file 9 — Additional file 9. Incidence rate per 100,000 person-years of malaria cases. According-to-protocol cohort, 6 to 12 weeks age-group [file 12936_2021_3670_MOESM9_ESM.docx]

Additional file 9 Incidence rate per 100,000 person-years of malaria cases. According-to-protocol cohort, 6 to 12 weeks age-group

|  | **Kombewa, Kenya** | | | **Kintampo, Ghana** | | | **Navrongo, Ghana** | | | **Overall** | | |
| --- | --- | --- | --- | --- | --- | --- | --- | --- | --- | --- | --- | --- |
|  | **N=1770** | | | **N=4120** | | | **N=958** | | | **N=6848** | | |
|  | **n** | **PY** | **Value (95% CI)** | **n** | **PY** | **Value (95% CI)** | **n** | **PY** | **Value (95% CI)** | **n** | **PY** | **Value (95% CI)** |
| **Malaria** |  |  |  |  |  |  |  |  |  |  |  |  |
| Any | 400 | 810 | 49,408  (44,684, 54,496) | 775 | 1963 | 39,480  (36,749, 42,360) | 35 | 443 | 7894  (5499, 10,979) | 1210 | 3216 | 37 625  (35,534, 39,806) |
| Uncomplicated | 393 | 810 | 48,544  (43,862, 53,589) | 754 | 1963 | 38,410  (35,717, 41,252) | 35 | 443 | 7894  (5499, 10,979) | 1182 | 3216 | 36 754  (34,688, 38,911) |
| Severe | 7 | 810 | 865  (348, 1782) | 21 | 1963 | 1070  (662, 1635) | 0 | 443 | 0  (0, 832) | 28 | 3216 | 871  (579, 1258) |
|  |  |  |  |  |  |  |  |  |  |  |  |  |
| ***P.* *falciparum*** |  |  |  |  |  |  |  |  |  |  |  |  |
| Uncomplicated | 369 | 810 | 45,579  (41,047, 50,475) | 629 | 1963 | 32,042  (29,587, 34,647) | 35 | 443 | 7894  (5499, 10,979) | 1,033 | 3216 | 32,121  (30,192, 34,141) |
| Severe | 7 | 810 | 865  (348, 1782) | 21 | 1963 | 1070  (662, 1635) | 0 | 443 | 0  (0, 832) | 28 | 3216 | 871  (579, 1258) |

N, Number of study participants at risk during a follow-up period of approximately 6 months after the 3^rd^ DTP-HepB-Hib vaccine dose; n, number of cases reported during that follow-up period; 95% CI, 95% confidence interval; PY, person-years; CI confidence intervals. No case of cerebral malaria was reported in the 6 to -12 weeks age group.

Malaria cases were confirmed by rapid diagnostic test and/or microscopy.

References

1. **WHO case definition, 2003. WHO Coordinated Invasive Bacterial Vaccine Preventable Diseases (IB-VPD) Surveillance Network: Tier 1 Meningitis Surveillance.** [**https://www.who.int/immunization/monitoring_surveillance/resources/IB-VPD_Case_Defs.pdf**](https://www.who.int/immunization/monitoring_surveillance/resources/IB-VPD_Case_Defs.pdf) **Accessed 26 February 2019.**

2. **World Health Organization. Guidelines for the treatment of malaria. 3rd Edition. 2015.** [**http://www.who.int/malaria/publications/atoz/9789241549127/en/**](http://www.who.int/malaria/publications/atoz/9789241549127/en/)**. Accessed 26 February 2019.**
