# Supplementary material for: Baseline incidence of meningitis, malaria, mortality and other health outcomes in infants and young sub-Saharan African children prior to the introduction of the RTS,S/AS01E malaria vaccine
Source: Malar J. 2021 Apr 26;20:197. doi: 10.1186/s12936-021-03670-w (PMC8073890; doi:10.1186/s12936-021-03670-w)
Supplement: Supplementary file 10 — Additional file 10. All-cause mortality rate per 100,000 person-years by gender. According-to protocol-cohort, 6 to 12 weeks age-group [file 12936_2021_3670_MOESM10_ESM.docx]

Additional file 10 All-cause mortality rate per 100,000 person-years by gender. According-to protocol-cohort, 6 to -12 weeks age-group

|  | **Kombewa, Kenya** | | | **Kintampo, Ghana** | | | **Navrongo, Ghana** | | | **Overall** | | |
| --- | --- | --- | --- | --- | --- | --- | --- | --- | --- | --- | --- | --- |
|  | **n** | **PY** | **Value (95% CI)** | **n** | **PY** | **Value (95% CI)** | **n** | **PY** | **Value (95% CI)** | **n** | **PY** | **Value (95% CI)** |
| **Female** |  |  |  |  |  |  |  |  |  |  |  |  |
| All causes | 6 | 579 | 1035 (380, 2254) | 16 | 1376 | 1162 (664, 1888) | 4 | 314 | 1272 (347, 3258) | 26 | 2270 | 1145 (748, 1678) |
| AESI | 0 | 579 | 0 (0, 634) | 0 | 1376 | 0 (0, 268) | 0 | 314 | 0 (0, 1174) | 0 | 2270 | 0 (0, 162) |
| Meningitis | 0 | 579 | 0 (0, 634) | 0 | 1376 | 0 (0, 268) | 0 | 314 | 0 (0, 1174) | 0 | 2270 | 0 (0, 162) |
| Malaria | 1 | 579 | 173 (4, 962) | 4 | 1376 | 291 (79, 744) | 1 | 314 | 318 (8, 1772) | 6 | 2270 | 264 (97, 575) |
| *P. falciparum* | 0 | 579 | 0 (0, 634) | 0 | 1376 | 0 (0, 268) | 0 | 314 | 0 (0, 1174) | 0 | 2270 | 0 (0, 162) |
| Other AEs | 5 | 579 | 863 (280, 2014) | 12 | 1376 | 872 (450, 1523) | 3 | 314 | 954 (197, 2789) | 20 | 2270 | 881 (538, 1361) |
| **Male** |  |  |  |  |  |  |  |  |  |  |  |  |
| All causes | 10 | 618 | 1618 (776, 2976) | 8 | 1439 | 556 (240, 1096) | 3 | 285 | 1054 (217, 3080) | 21 | 2341 | 897 (555, 1371) |
| AESI | 0 | 618 | 0 (0, 597) | 0 | 1439 | 0 (0, 256) | 0 | 285 | 0 (0, 1296) | 0 | 2341 | 0 (0, 158) |
| Meningitis | 1 | 618 | 162 (4, 902) | 0 | 1439 | 0 (0, 256) | 0 | 285 | 0 (0, 1296) | 1 | 2341 | 43 (1, 238) |
| Malaria | 5 | 618 | 809 (263, 1888) | 0 | 1439 | 0 (0, 256) | 0 | 285 | 0 (0, 1296) | 5 | 2341 | 214 (69, 498) |
| *P. falciparum* | 2 | 618 | 324 (39, 1169) | 0 | 1439 | 0 (0, 256) | 0 | 285 | 0 (0, 1296) | 2 | 2341 | 85 (10, 309) |
| Other AEs | 4 | 618 | 647 (176, 1658) | 8 | 1439 | 556 (240, 1096) | 3 | 285 | 1054 (217, 3080) | 15 | 2341 | 641 (359, 1057) |
|  |  |  |  |  |  |  |  |  |  |  |  |  |
| **Total** |  |  |  |  |  |  |  |  |  |  |  |  |
| All causes | 16 | 1197 | 1336 (764, 2170) | 24 | 2815 | 853 (546, 1268) | 7 | 599 | 1169 (470, 2408) | 47 | 4611 | 1019 (749, 1355) |
| AESI | 0 | 1197 | 0 (0, 308) | 0 | 2815 | 0 (0, 131) | 0 | 599 | 0 (0, 616) | 0 | 4611 | 0 (0, 80) |
| Meningitis | 1 | 1197 | 84 (2, 465) | 0 | 2815 | 0 (0, 131) | 0 | 599 | 0 (0, 616) | 1 | 4611 | 22 (1, 121) |
| Malaria | 6 | 1197 | 501 (184, 1091) | 4 | 2815 | 142 (39, 364) | 1 | 599 | 167(4, 930) | 11 | 4611 | 239 (119, 427) |
| *P. falciparum* | 2 | 1197 | 167 (20, 603) | 0 | 2815 | 0 (0, 131) | 0 | 599 | 0 (0, 616) | 2 | 4611 | 43 (5, 157) |
| Other AEs | 9 | 1197 | 752 (344, 1427) | 20 | 2815 | 710 (434, 1097) | 6 | 599 | 1002 (368, 2180) | 35 | 4611 | 759 (529, 1056) |

N, Number of study participants at risk during a follow-up period of approximately 6 months after each dose of the DTP-HepB-Hib primary vaccination schedule censored at the next dose; n, number of cases reported during that follow-up period; PY, person-years; CI, confidence interval; AE, adverse event; AESI, adverse events of special interest.
