# Supplementary material for: Baseline incidence of meningitis, malaria, mortality and other health outcomes in infants and young sub-Saharan African children prior to the introduction of the RTS,S/AS01E malaria vaccine
Source: Malar J. 2021 Apr 26;20:197. doi: 10.1186/s12936-021-03670-w (PMC8073890; doi:10.1186/s12936-021-03670-w)
Supplement: Supplementary file 11 — Additional file 11. Incidence rate per 100,000 person-years of other adverse events leading to hospitalization. ATP, 6 to 12 weeks age-group [file 12936_2021_3670_MOESM11_ESM.docx]

Additional file 11 Incidence rate per 100,000 person-years of other adverse events leading to hospitalization. ATP, 6 to 12 weeks age-group

|  | **Kombewa, Kenya** | | | | **Kintampo, Ghana** | | | | **Navrongo, Ghana** | | | | **Overall** | | | |
| --- | --- | --- | --- | --- | --- | --- | --- | --- | --- | --- | --- | --- | --- | --- | --- | --- |
|  | **N=1979** | | | | **N=4297** | | | | **N=972** | | | | **N=7248** | | | |
|  | **n** | **PY** | **Value (95% CI)** | | **n** | **PY** | **Value (95% CI)** | | **n** | **PY** | **Value (95% CI)** | | **n** | **PY** | **Value (95% CI)** | |
| Anemia | 6 | 433 | 1385 | (508, 3015) | 52 | 1018 | 5107 | (3814, 6697) | 18 | 224 | 8044 | (4767, 12 713) | 76 | 1675 | 4537 | (3575, 5679) |
| Bacterial Infection | 2 | 433 | 462 | (56, 1668) | 0 | 1020 | 0 | (0, 362) | 0 | 224 | 0 | (0, 1644) | 2 | 1678 | 119 | (14, 431) |
| Burns | 0 | 433 | 0 | (0, 851) | 0 | 1020 | 0 | (0, 362) | 0 | 224 | 0 | (0, 1644) | 0 | 1678 | 0 | (0, 220) |
| Conjunctivitis | 0 | 433 | 0 | (0, 851) | 1 | 1020 | 98 | (2, 546) | 0 | 224 | 0 | (0, 1644) | 1 | 1678 | 60 | (2, 332) |
| Gastroenteritis | 2 | 433 | 462 | (56, 1668) | 18 | 1020 | 1765 | (1046, 2790) | 3 | 224 | 1337 | (276, 3909) | 23 | 1677 | 1371 | (869, 2058) |
| Helminthic Infection | 0 | 433 | 0 | (0, 851) | 0 | 1020 | 0 | (0, 362) | 0 | 224 | 0 | (0, 1644) | 0 | 1678 | 0 | (0, 220) |
|  |  |  |  |  |  |  |  |  |  |  |  |  |  |  |  |  |
| Lower Respiratory Tract Infection | 5 | 433 | 1154 | (375, 2694) | 36 | 1019 | 3533 | (2475, 4891) | 6 | 224 | 2677 | (982, 5827) | 47 | 1676 | 2804 | (2060, 3729) |
| Malnutrition | 0 | 433 | 0 | (0, 851) | 0 | 1020 | 0 | (0, 362) | 1 | 224 | 446 | (11, 2484) | 1 | 1678 | 60 | (2, 332) |
| Sepsis | 0 | 433 | 0 | (0, 851) | 13 | 1020 | 1275 | (679, 2181) | 3 | 224 | 1338 | (276, 3911) | 16 | 1677 | 954 | (545, 1549) |
| Skin Infection | 1 | 433 | 231 | (6, 1286) | 7 | 1020 | 686 | (276, 1414) | 2 | 224 | 892 | (108, 3222) | 10 | 1677 | 596 | (286, 1096) |
| Upper respiratory tract infection | 0 | 433 | 0 | (0, 851) | 4 | 1020 | 392 | (107, 1004) | 3 | 224 | 1337 | (276, 3909) | 7 | 1678 | 417 | (168, 860) |
| Urinary Tract Infection | 0 | 433 | 0 | (0, 851) | 0 | 1020 | 0 | (0, 362) | 0 | 224 | 0 | (0, 1644) | 0 | 1678 | 0 | (0, 220) |

Preferred terms of other AEs leading to hospitalization were grouped into medically relevant categories (refer to Table S2 of the supplement for more details) for the calculation on the incidence rates. N, Number of study participants at risk within 30 days after each dose of the DTP-HepB-Hib primary vaccination schedule censored at the next dose; n, number of cases reported during that follow-up period; PY, person-years; CI, confidence interval; ATP, According-to protocol-cohort.
