# Supplementary material for: Baseline incidence of meningitis, malaria, mortality and other health outcomes in infants and young sub-Saharan African children prior to the introduction of the RTS,S/AS01E malaria vaccine
Source: Malar J. 2021 Apr 26;20:197. doi: 10.1186/s12936-021-03670-w (PMC8073890; doi:10.1186/s12936-021-03670-w)
Supplement: Supplementary file 12 — Additional file 12. Incidence rate of other adverse events leading to hospitalization, by study site. ATP, 5 to 17 months age-group [file 12936_2021_3670_MOESM12_ESM.docx]

Additional file 12 Incidence rate of other adverse events leading to hospitalization, by study site. ATP, 5 to 17 months age-group

|  | **Kombewa, Kenya** | | | | **Kintampo, Ghana** | | | | **Navrongo, Ghana** | | | |
| --- | --- | --- | --- | --- | --- | --- | --- | --- | --- | --- | --- | --- |
|  | **N=2111** | | | | **N=3603** | | | | **N=1183** | | | |
|  | **n** | **PY** | **Value (95% CI)** | | **n** | **PY** | **Value (95% CI)** | | **n** | **PY** | **Value (95% CI)** | |
| Anemia | 7 | 411 | 1705 | (685, 3513) | 41 | 812 | 5050 | (3624, 6851) | 21 | 267 | 7858 | (4864, 12,012) |
| Bacterial Infection | 0 | 411 | 0 | (0, 898) | 0 | 814 | 0 | (0, 453) | 1 | 268 | 373 | (9, 2080) |
| Burns | 1 | 411 | 243 | (6, 1357) | 0 | 814 | 0 | (0, 453) | 1 | 268 | 373 | (9, 2080) |
| Conjunctivitis | 0 | 411 | 0 | (0, 898) | 1 | 814 | 123 | (3, 685) | 0 | 268 | 0 | (0, 1377) |
| Gastroenteritis | 6 | 411 | 1461 | (536, 3181) | 32 | 812 | 3940 | (2695, 5562) | 3 | 268 | 1120 | (231, 3273) |
| Helminthic Infection | 0 | 411 | 0 | (0, 898) | 2 | 813 | 246 | (30, 888) | 0 | 268 | 0 | (0, 1377) |
| Lower Respiratory Tract Infection | 5 | 411 | 1218 | (395, 2842) | 29 | 812 | 3570 | (2391, 5127) | 4 | 268 | 1493 | (407, 3824) |
| Malnutrition | 1 | 411 | 243 | (6, 1356) | 2 | 813 | 246 | (30, 888) | 0 | 268 | 0 | (0, 1377) |
| Sepsis | 2 | 411 | 487 | (59, 1759) | 18 | 813 | 2215 | (1313, 3500) | 2 | 268 | 747 | (90, 2697) |
| Skin Infection | 0 | 411 | 0 | (0, 898) | 3 | 813 | 369 | (76, 1078) | 0 | 268 | 0 | (0, 1377) |
| Upper respiratory tract infection | 5 | 411 | 1218 | (395, 2841) | 9 | 813 | 1107 | (506, 2101) | 5 | 268 | 1867 | (606, 4357) |
| Urinary Tract Infection | 0 | 411 | 0 | (0, 898) | 0 | 814 | 0 | (0, 453) | 0 | 268 | 0 | (0, 1377) |

Incidence rate per 100,000 person-years. Preferred terms of other AEs leading to hospitalization were grouped into medically relevant categories (refer to Table S2 of the supplement for more details) for the calculation on the incidence rates. N, Number of study participants at risk within 30 days after each dose of the virtual primary vaccination schedule censored at the next dose; n, number of cases reported during that follow-up period; CI, confidence interval; PY, person-years; ATP, According-to protocol-cohort.
